# Elimination Reaction-Based Benzimidazole Probe for Cysteine Detection and Its Application in Serum Sample Analysis

**DOI:** 10.3390/bios12040224

**Published:** 2022-04-08

**Authors:** In-ho Song, Gyu Seong Yeom, Anil Kuwar, Satish Balasaheb Nimse

**Affiliations:** 1 Institute of Applied Chemistry and Department of Chemistry, Hallym University, Chuncheon 24252, Korea; songin-ho@hallym.ac.kr (I.-h.S.); m21019@hallym.ac.kr (G.S.Y.); 2 School of Chemical Sciences, KBC-North Maharashtra University, Jalgaon 425001, India; kuwaras@gmail.com

**Keywords:** biothiols, cysteine, fluorescence, leukemia, Michael addition, serum

## Abstract

Benzimidazole-based compound 2-(*p*-tolyl)-1*H*-benzo[d]imidazole (**3**) and its derivative probe **A-B** have been synthesized for the highly selective detection and quantification of Cys in human serum. The photophysical properties of **A-B** and compound **3** were evaluated by UV-vis absorption and fluorescence spectroscopy. **A-B** showed high selectivity and sensitivity for Cys among tested analytes, including amino acids, anions, and cations. **A-B** selectively reacts with Cys and results in compound **3** with fluorescence turn-on effect. **A-B** did not show any interference from the components in the serum matrix for Cys detection in the human serum sample. **A-B** detects Cys in serum samples with 2.3–5.4-fold better LOD than reported methods. The detection limit of 86 nM and 43 nM in HEPES buffer using UV-visible and fluorescence spectroscopy, respectively, makes **A-B** an excellent chemosensor for Cys detection.

## 1. Introduction

**Figure 1:**
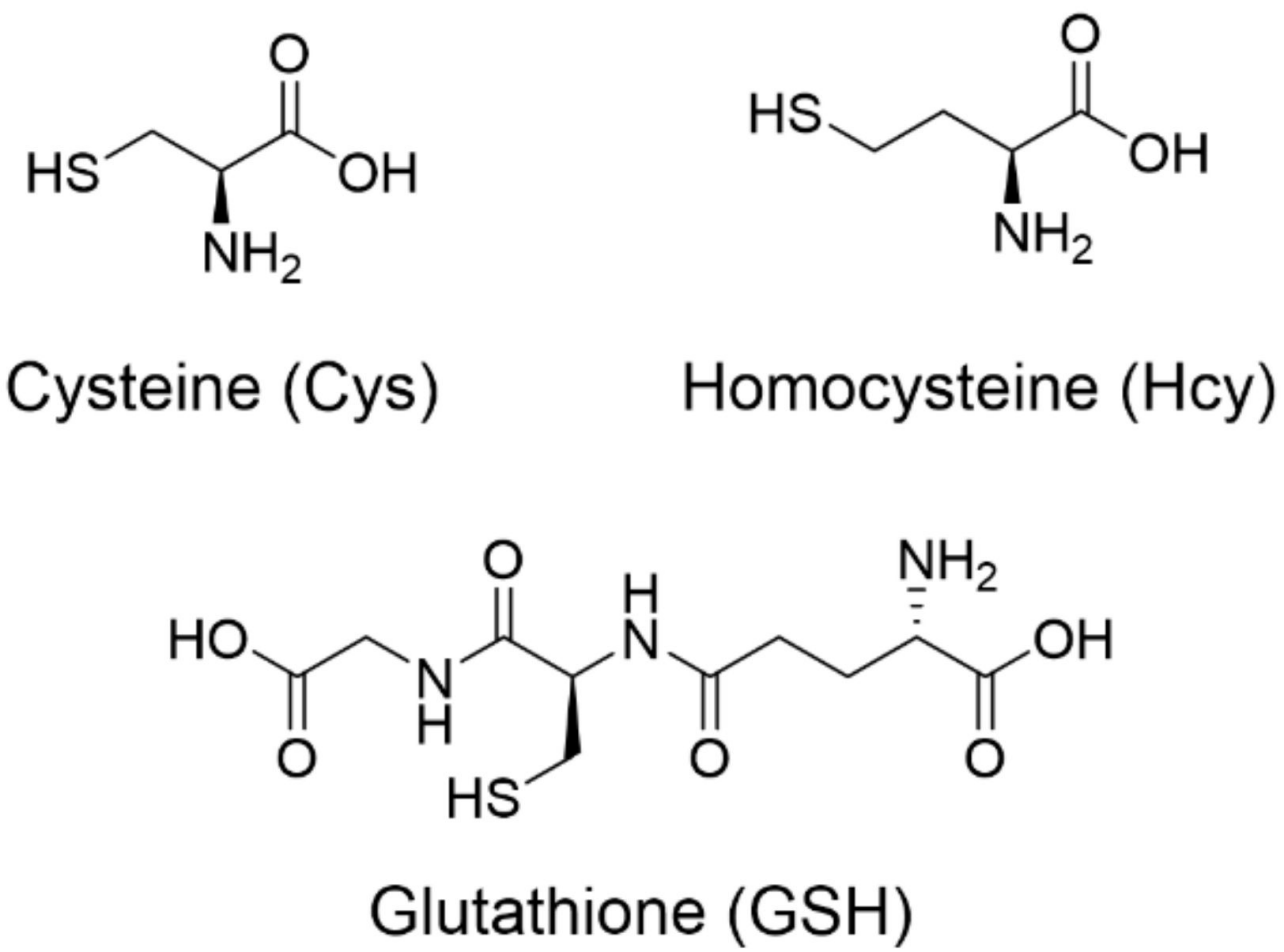
Important biothiols, including cysteine (Cys), homocysteine (Hcy), and glutathione (GSH) present in serum.

The sulfhydryl or thiol functional (-SH) group in biomolecules has long been known to participate in crucial biochemical reactions in almost all living species [1,2]. The biothiols present as individual molecules or parts of peptides and proteins that are key reactive sulfur species (RSS) required for normal functions in the human body [3,4,5,6]. The biothiols mainly include cysteine (Cys), glutathione (GSH), and homocysteine (Hcy) (Figure 1). These biothiols play a significant role in antioxidants by protecting cells from being tarnished by free radicals [7,8,9].

In particular, the lack of Cys affects growth in children, liver function, muscle loss, leukemia, and arteriosclerosis [10,11,12]. Moreover, Cys proficiently binds with metal ions, including mercury, copper, lead, etc. [13,14,15]. Cys plays a crucial role in cancer cell metabolism, and hence it is present to a large extent in cancer cells compared to normal cells [16,17,18]. Therefore, detecting and quantifying Cys in biological samples such as serum is crucial for identifying several disorders and diseases, such as muscle and fat loss, edema, narcolepsy, and liver damage [19,20].

Several analytical methods, including gas chromatography and high-performance liquid chromatography, are currently used to monitor low levels of these biomolecules. However, these methods are time-consuming, expensive, and require highly trained professionals [21,22]. Therefore, techniques that have simple experimental procedures and allow the precise and successful detection of these biomolecules at low cost are in high demand. Among fluorescent chemosensors, near-infrared (NIR) fluorescence probes are developed for imaging applications to detect Cys in various tissues, including tumors [23]. The NIR chemosensors often possess complex structures and require multistep reactions to afford them [24,25,26]. Though NIR chemosensors are excellent, a simple but highly specific fluorescence turn-on probe is advantageous for detecting Cys in body fluids, including serum.

There are several reports on benzimidazole derivatives as chemosensors for detecting various analytes, including peroxynitrite (ONOO^−^), Fe^3+^, Al^3+^, and Zn^2+^ [27,28,29,30,31,32]. However, there are no reports on the application of benzimidazole derivatives to develop a chemosensor for detecting Cys in human serum samples. The benzimidazole chromophore demonstrates fluorescence properties that, with proper modifications, can be harnessed for detecting the analytes of interest [33]. The acrylate functional group substitution on the phenolic hydroxyl groups has been used extensively in the probes used to detect biothiols [34,35,36,37]. The acrylate-substituted fluorophores lose their usual fluorescence because of intermolecular charge transfer (ICT) [38,39,40]. The ICT happens from the fluorophore to the acrylate functional group. However, upon adding biothiols, the acrylate group is eliminated through a cascade of addition, cyclization, and elimination reactions liberating the original fluorophore that now shows significant fluorescence enhancement. To the best of our knowledge, the fluorescence turn-on probe based on the substitution of acrylate functional group on benzimidazole nitrogen has not been studied widely.

Here, we report the synthesis and characterization of 2-(*p*-tolyl)-1H-benzo[d]imidazole (compound **3**) and its successful modification into a 1-(2-(*p*-tolyl)-1H-benzo[d]imidazol-1-yl)prop-2-en-1-one (probe **A-B**) for the highly selective and sensitive detection of Cys in human serum. With only two design elements that included a benzimidazole ring system for fluorescence and an acrylate functional group as a recognition moiety, **A-B** is one of the simplest fluorescent probes developed for the highly sensitive detection of Cys. We investigated the feasibility of the benzimidazole-based fluorescence turn-on probe **A-B** for detecting biothiols in the aqueous system and the spiked human serum samples. **A-B** with a quantum yield of 0.69 and almost no interference from the components in the serum matrix demonstrated high applicability for Cys detection in serum samples. The lower detection limit using UV−vis (86 nM) and fluorescence spectroscopy (43 nM) makes **A-B** an excellent chemosensor for Cys detection. **A-B** was evaluated for detecting and quantifying Cys in spiked human serum samples. The obtained results indicate the high applicability of **A-B** in clinical settings for Cys measurements in serum samples.

## 2. Material and Methods

### 2.1. Reagents and Instruments

Required reagents were procured from Sigma-Aldrich (Seoul, South Korea) and used as received unless otherwise stated. The thin-layer chromatography (TLC) plates (Silica Gel 60 F254) were procured from Merck (Seoul, South Korea). The developed plates were visualized under UV light (254 nm). Normal human serum (90R-1002) was procured from Fitzgerald Industries International, USA. The ^1^H and ^13^C NMR were recorded on a Jeol FT-NMR spectrometer (400 MHz; JEOL, Akishima, Tokyo, Japan). Shimadzu UV-1800 spectrometer (Shimadzu, Kyoto, Japan) was used for the UV−vis measurements. Agilent Cary Eclipse fluorescence spectrophotometer (Agilent Technologies, Santa Clara, CA, USA) was used for the fluorescence measurements. JMS-700 MStation Mass Spectrometer (JEOL, Akishima, Tokyo, Japan) was used to measure high-resolution mass spectra. We measured the pH of solutions with a STARTER 300 portable pH meter (Ohaus Corporation, Parsippany, Morris, NJ, USA).

### 2.2. Synthesis of 2-(p-Tolyl)-1H-benzo[d]imidazole *(**3**)*

A solution of 4-methylbenzaldehyde (0.51 g, 4.24 mmol) in ethanol (5 mL) was added dropwise for 2 h to the ethanolic solution (15 mL) of benzene-1,2-diamine (0.46 g, 4.24 mmol) with a dropping funnel. Then, we continued to stir the reaction mixture under air at 25 °C and the reaction progress was monitored by TLC (ethyl acetate:hexanes, 3:7 mixture). The precipitated product was filtered after completion of reaction. The collected product was washed three times with ethanol (10 mL) and vacuum dried to obtain the final product. White-colored powder: yield: 0.70 g (79%). mp 275 °C. ^1^H-NMR (400 MHz, DMSO-d_6_) δ 12.81 (s, 1H, -NH), 8.07 (d, *J* = 8.2 Hz, 2H, Ar-H), 7.64 (d, *J* = 6.9 Hz, 1H, Ar-H), 7.51 (dd, *J* = 6.7, 1.7 Hz, 1H, Ar-H), 7.36 (d, *J* = 7.9 Hz, 2H, Ar-H), 7.22–7.14 (m, 2H, Ar-H), 2.38 (s, 3H, -CH_3_). ^13^C NMR (100 MHz, DMSO-d_6_) δ 151.42, 139.57, 129.53, 127.49, 126.41, 39.52, 20.98; HR-EIMS^+^: m/z = 208.1000 [M]^+^. (Calcd. for C_14_H_12_N_2_, 208.10).

### 2.3. Synthesis 1-(2-(p-Tolyl)-1H-benzo[d]imidazol-1-yl)prop-2-en-1-one **(*A-B*)**

A solution of compound **3** (0.7 g, 3.36 mmol) and triethylamine (0.56 mL, 4.01 mmol) in anhydrous dichloromethane (20 mL) was stirred at 0 °C for 30 min. Then, prop-2-enoyl chloride (0.37 g, 4.01 mmol) was added and the reaction was continued until completion (TLC using ethyl acetate:hexanes, 3:7 mixture). After completion, the reaction mixture was washed three times with saturated Na_2_CO_3_ (50 mL) and one time with brine (50 mL). The separated organic layer was dried over anhydrous MgSO_4_. Finally, the crude product was subjected to purification by column chromatography (SiO2, ethyl acetate/hexane = 3:7, v/v) after evaporating the solvent. Final compound **A-B** was obtained as a white solid. Yield 0.68 g (77%). mp 122 °C. ^1^H-NMR (400 MHz, DMSO-d_6_) δ 8.17–7.99 (m, 1H, Ar-H), 7.86–7.75 (m, 1H, Ar-H), 7.62–7.53 (m, 2H, Ar-H), 7.43–7.37 (m, 2H, Ar-H), 7.32–7.27 (m, 2H, Ar-H), 6.56 (dd, *J* = 16.8, 1.2 Hz, 1H), 6.14 (dd, *J* = 16.8, 10.3 Hz, 1H), 5.74 (dd, *J* = 10.3, 1.2 Hz, 1H), 2.44 (s, 3H, -CH_3_). ^13^C NMR (100 MHz, Chloroform-d) δ 165.47, 153.08, 142.88, 141.20, 134.37, 132.42, 130.74, 129.76, 129.66, 128.25, 125.31, 125.10, 120.14, 114.59, 21.66. HR-EIMS^+^: m/z = 262.1106 [M]^+^. (Calcd. for C_17_H_14_N_2_O, 262.11).

### 2.4. Determination of Selectivity and Sensitivity of ***A-B***

The stock solution of **A-B** (10 mM) was prepared in spectroscopic grade DMSO. The working solution of **A-B** (10 µM) was prepared by diluting the stock solution with 1 mM HEPES buffer (pH = 7.4) such that the amount of DMSO was 0.1%. The stock solution of various analytes (10 mM), including amino acids, anions, and cations, was prepared in HEPES buffer. The selectivity of **A-B** was determined by adding the solutions of various analytes (5 equivalent, 50 µM) to the solution of **A-B**.

The studied analytes included essential amino acids such as histidine (His), isoleucine (Ile), leucine (Leu), lysine (Lys), methionine (Met), phenylalanine (Phe), threonine (Thr), tryptophan (Typ), and valine (Val). The non-essential amino acids included in the study were glycine (Gly), alanine (Ala), serine (Ser), proline (Pro), asparagine (Asp), aspartic acid (Asn), glutamine (Gln), glutamic acid (Glu), arginine (Arg), tyrosine (Tyr), homocysteine (Hcy), glutathione (GSH), and cysteine (Cys). We also included the other biologically relevant ions such as Cl^−^, Br^−^, NO_3_^−^, AcO^−^, HSO_4_^2−^, H_2_PO_4_^−^, Na^+^, Cs^+^, Ca^2+^, and Cu^2+^.

The sensitivity of **A-B** for detection of Cys was studied by stepwise addition of 1.6 equivalents Cys (1 mM) to the solution of **A-B** (10 µM). The absorbance and emission spectra were recorded in the range of 230–600 and 300–650 nm, respectively, alongside a reagent blank. The fluorescence intensity was recorded at λ_ex_/λ_em_ = 303/350 nm (excitation slit = 5 nm, emission slit = 5.0 nm). Experiments were performed in triplicate. We used the mean and standard deviation values for further analysis. The kinetic parameter such as rate constant (*k*) was determined by monitoring the changes in fluorescence intensity with respect to time (0 to 30 min) upon addition of 5 equivalent Cys (50 µM) in the solution of **A-B** (10 µM). The rate constant was calculated using the following equation (Equation (1)) as the reaction of Cys with **A-B** follows pseudo-first-order kinetics [41].

(1) $$\ln(\left(I_{max}-I_{t}\right)/I_{max})=-kt$$

*I_t_* is the fluorescence intensity at time *t*, *I_max_* is the fluorescence intensity upon reaction, and *k* is the rate constant.

### 2.5. Effect of pH on Cys Detection and Quantum Yield Compound ***3***

Fluorescence spectra were recorded to investigate the effect of pH (pH = 2–12) on the detection of Cys using **A-B**. The tetrabutylammonium hydroxide and perchloric acid were used to adjust the pH of the solution.

The quantum yield (Φ) of compound **3** was measured using the following formula (Equation (2)).

(2) $$\Phi_{\mathrm{sample}}=\left\{\frac{\left({\mathrm{OD}}_{\mathrm{standard}}\times A_{\mathrm{sample}}\times \eta_{\mathrm{sample}}^{2}\right)}{\left({\mathrm{OD}}_{\mathrm{sample}}\times A_{\mathrm{standard}}\times \eta_{\mathrm{standard}}^{2}\right)}\right\}\Phi_{\mathrm{standard}}$$

where A is the area under the emission spectral curve, OD is the compound’s optical density at the excitation wavelength, and *η* is the refractive index of the solvent.

### 2.6. Determination of Limit of Detection (LOD)

We used the following IUPAC-recommended equation (Equation (3)) to estimate the LOD for Cys detection by **A-B** [42].

(3) $$LOD=\frac{\sigma }{m}$$

where *σ* is the standard deviation of blank samples (n = 10) and *m* is the slope of a calibration curve.

### 2.7. Determination of Cysteine in Serum Sample

We used the standard addition method to quantify Cys in serum samples to minimize the interference from serum matrix components. We used the spiked serum samples to validate the presented method through the standard addition method due to the unavailability of a commercial cysteine detection kit. The serum sample aliquots were prepared after thawing the serum at 25 °C for 30 min. About 2 µL of these serum samples were mixed with 1 mL of **A-B** (10 µM) solution in HEPES buffer, and the fluorescence/UV spectra were measured. The fluorescence spectra were recorded by setting 303 nm and 350 nm as excitation and emission wavelengths. Then, the serum samples were spiked with cysteine to obtain final concentrations of 2, 4, and 6 µM. The experiment was performed in triplicate for each sample, and the concentrations of spiked samples were determined using the standard curve.

## 3. Results and Discussion

### 3.1. Synthesis and Characterization of Compound ***3*** and Probe ***A-B***

**Scheme 1:**
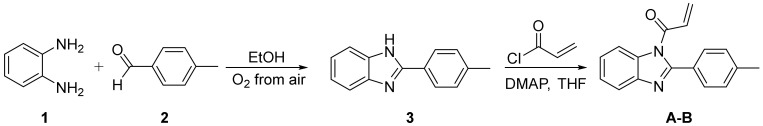
Scheme for the synthesis of the probe **A-B**.

Probe **A-B** was synthesized as depicted in Scheme 1. In brief, the benzene-1,2-diamine (4.24 mmol) and 4-methylbenzaldehyde (4.24 mmol) underwent reaction in ethanol at 25 °C. The reaction was carried out under visible light in the presence of O_2_ from the air. These mild reaction conditions allowed us to afford compound **3** with a 79% yield (see the Supplementary Materials Figures S1–S3).

The spectral investigation by ^1^H-NMR, ^13^C-NMR, and mass spectroscopic methods gave consistent data for the molecular structure of compound **3**. The reported procedures employed for synthesizing benzimidazole derivatives are different from the method used here [43,44]. The use of stringent reaction conditions, including the reaction temperature of 110 °C, dimethyl sulfoxides as a solvent, and I_2_ as a catalyst, is reported for the synthesis of benzimidazole derivatives. The method employed here allowed synthesizing benzimidazole derivative **3** in high yield using mild reaction conditions. The ^1^H-NMR, ^13^C-NMR of **3** are consistent with literature data [45,46,47]. The reaction of compound **3** with the prop-2-enoyl chloride (4.01 mmol) in the presence of triethylamine (4.01 mmol) allows the synthesis of **A-B** (see the Supplementary Materials Figures S4–S6) in comparable yield.

**Figure 2:**
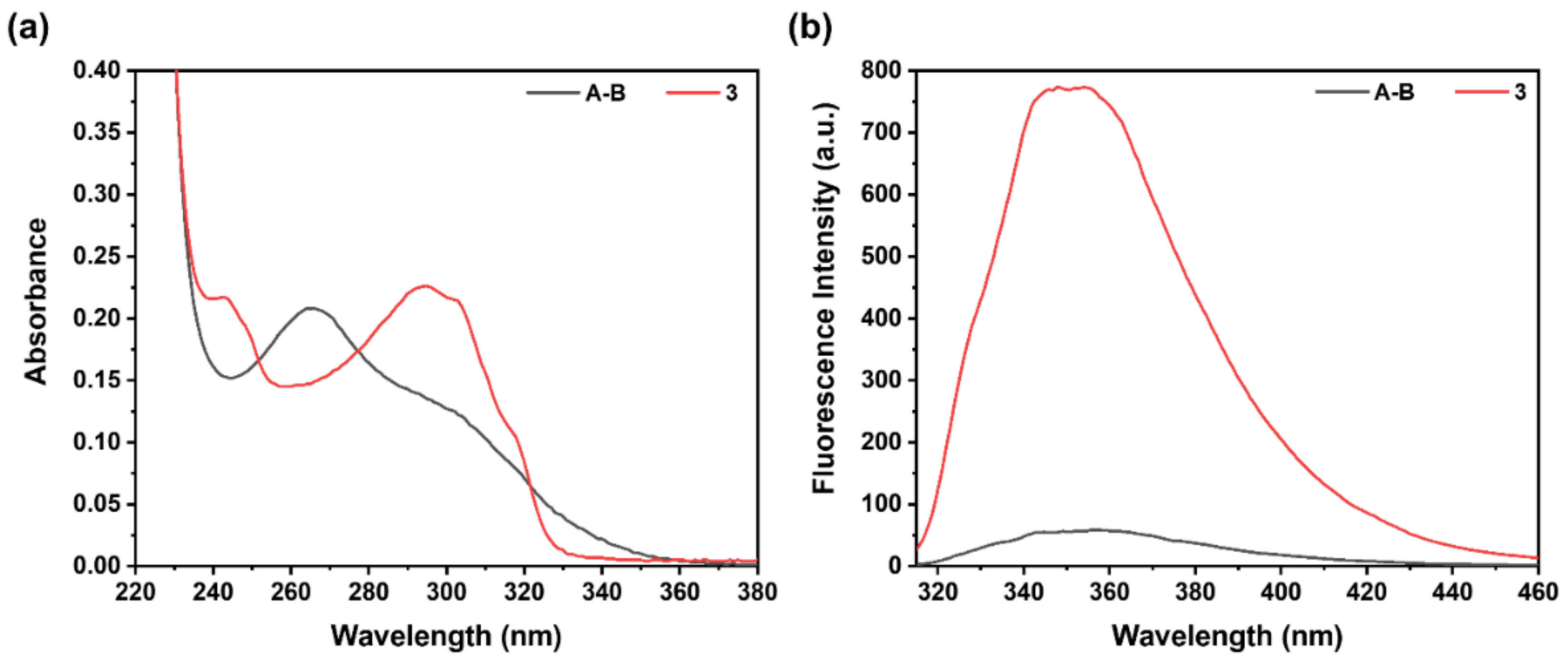
Characterization of compound **3** (10 µM; λ_ex_ = 303 nm, λ_em_ = 350 nm) and **A-B** (10 µM; λ_ex_ = 265 nm, λ_em_ = 357 nm) dissolved in 1 mM HEPES buffer solution containing 0.1% DMSO by (**a**) UV−Vis and (**b**) fluorescence emission spectra at room temperature.

The UV−vis and fluorescence spectra of compound **3** (10 µM) and **A-B** (10 µM) were recorded. As shown in Figure 2a, compound **3** showed an absorption band at 303 nm, most likely due to the n to π * transition. **A-B** exhibits peak maxima at 265 nm. The longer wavelength band at 265 nm may be assigned to transitions associated with the benzimidazole ring. As shown in Figure 2b, compound **3** demonstrated strong fluorescence with the emission maxima at 350 nm with a redshift (∆λ ≈ 47 nm). However, **A-B** exhibited mild fluorescence at the same concentration as compound **3**. The emission maxima for **A-B** were observed at 357 nm with a redshift of 92 nm. Though **A-B** showed a longer redshift, the observed fluorescence quenching compared to the fluorescence intensity of compound **3** is attributed to the acrylate group substitution on the benzimidazole nitrogen. A highly fluorescent compound **3**, upon reaction with the acryloyl chloride, results in the non-fluorescent **A-B**. The modulation of the benzimidazole π-electron system by conjugating the acrylate group to the ring nitrogen through amide bond activates an ICT responsible for the resulting fluorescence turn-off effect. These results indicate that removing the acrylate group via reaction with the biothiols can generate compound **3** with the appearance of fluorescence turn-on effect. Therefore, **A-B** has high applicability in detecting analytes due to its possible reaction-induced fluorescence turn-on effect.

### 3.2. **A-B** Is Highly Selective for Cys

**Figure 3:**
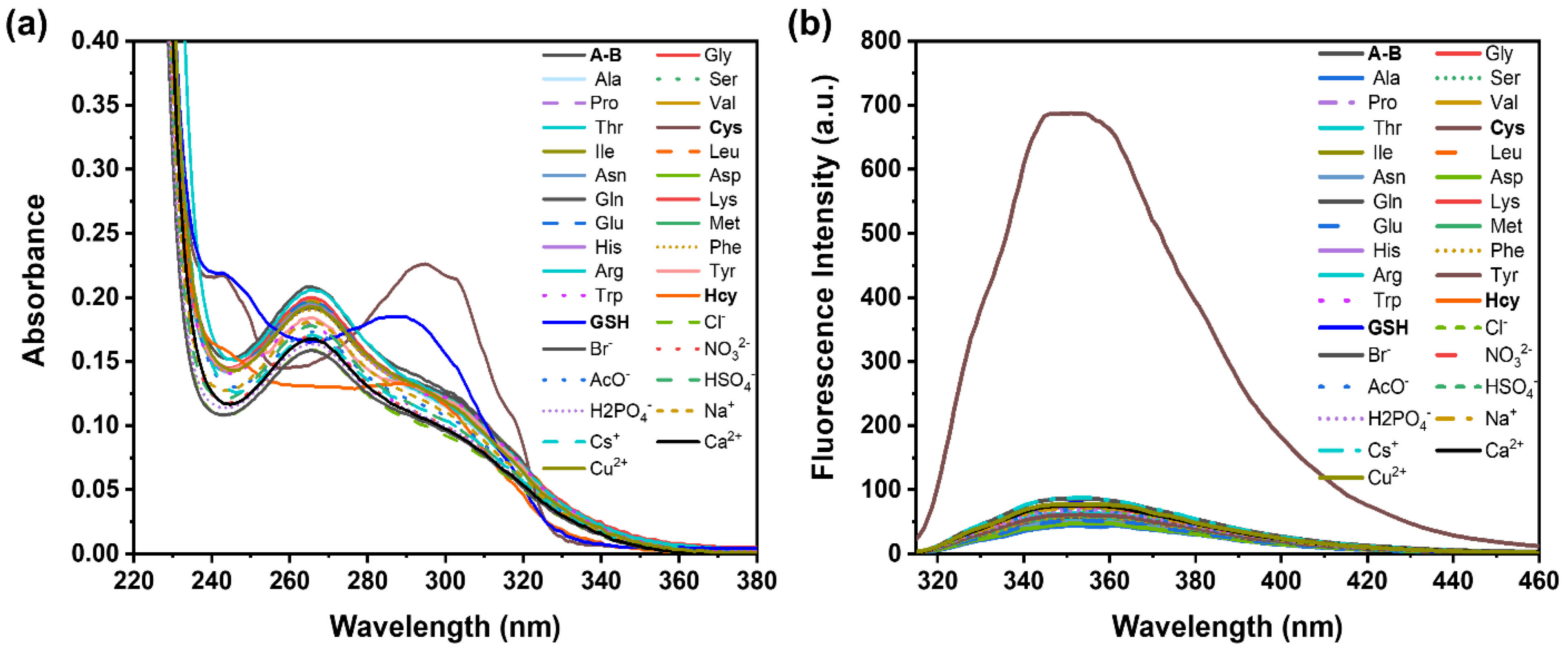
(**a**) Changes in UV−vis absorption spectra and (**b**) fluorescent intensity (λ_ex_ = 303 nm, λ_em_ = 350 nm) of **A-B** (10 µM) upon addition of 5 equivalents of other analytes (50 µM) in 1 mM HEPES buffer at room temperature.

After synthesis and characterization, **A-B** was applied for the selective optical sensing of a series of amino acids, anions, and cations in HEPES buffer (pH = 7.4). The absorption and emission spectrum of **A-B** (10 µM) in the absence and presence of various analytes (50 µM) was detected. As shown in Figure 3, probe **A-B** demonstrated excellent selectivity towards Cys compared to any other tested analytes (see the Supplementary Materials Figure S7). About 5 equivalents of analytes (50 µM) were added to the solutions of **A-B** (10 µM). Then, the UV−vis absorption and fluorescence spectra were recorded after 10 min incubation at room temperature.

As shown in Figure 3a, the absorption band of **A-B** at 265 nm was redshifted to 303 nm (∆λ ≈ 38 nm) upon reaction with Cys, indicating that **A-B** has a higher binding affinity towards Cys than other surveyed amino acids, anions, and cations. In the presence of other amino acids, **A-B** showed either no change or a moderate decrease in the absorption intensity. The possible reaction between Cys and **A-B** modulates the ICT state’s push-pull character, evidenced by the resultant redshift (∆λ ≈ 38 nm) in the absorption spectra. On the other hand, the redshifted absorption spectrum of **A-B** (Figure 3a) in the presence of Cys overlaps with the photon absorbance spectrum of compound **3** (Figure 2a), indicating the reestablishment of benzimidazole π-electron system.

Apart from UV−vis absorption spectroscopy measurements, the selectivity of **A-B** towards Cys was also established by fluorescence spectroscopy measurements. The fluorescence spectra of **A-B** (10 µM) were recorded with and without various analytes (50 µM) as depicted in Figure 3b. Upon excitation at 303 nm, **A-B** showed a strong emission peak at 350 nm in the presence of Cys. In contrast, none of the other analytes showed any change in the fluorescence signals, indicating that they did not show any interaction with **A-B**. These results suggested the modulation of the benzimidazole π-electron system due to the removal of acrylate group from **A-B** by possible reaction of –SH in Cys and acrylate results in the release of chromophore evidenced by the fluorescence turn-on effect. In short, the addition of Cys followed by elimination of the acrylate group in **A-B** modulates the “push-pull” property, leading to a better donor-acceptor system with observed fluorescence emission from the benzimidazole ring. These results indicated that **A-B** shows selectivity for Cys in both UV−visible and fluorescence spectroscopy measurements.

**Scheme 2:**
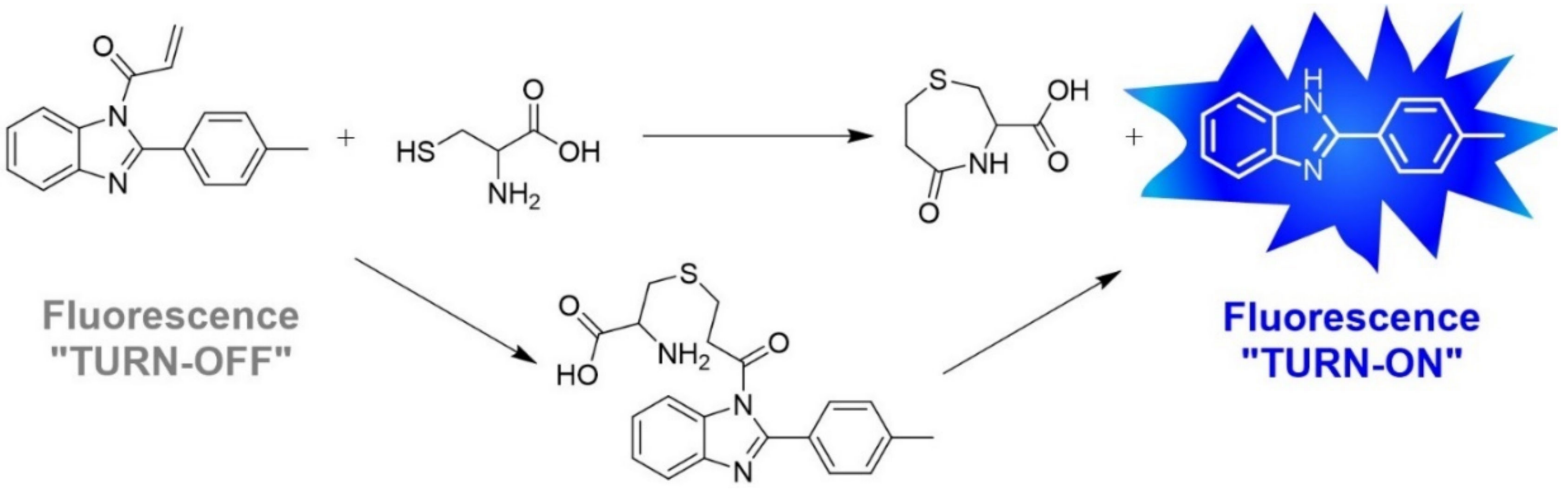
The recognition procedure of **A-B** towards cysteine.

Scheme 2 depicts the proposed fluorescence turn-on mechanism upon the reaction of Cys with **A-B**. The acrylate functional group on the benzimidazole ring regulates the π-electron system and triggers the ICT-based fluorescence turn-on effect. However, Michael addition of -SH group in Cys to the acrylate of **A-B** followed by elimination of a seven-membered ring disrupts the ICT process and results in the fluorescence turn-on effect.

**Figure 4:**
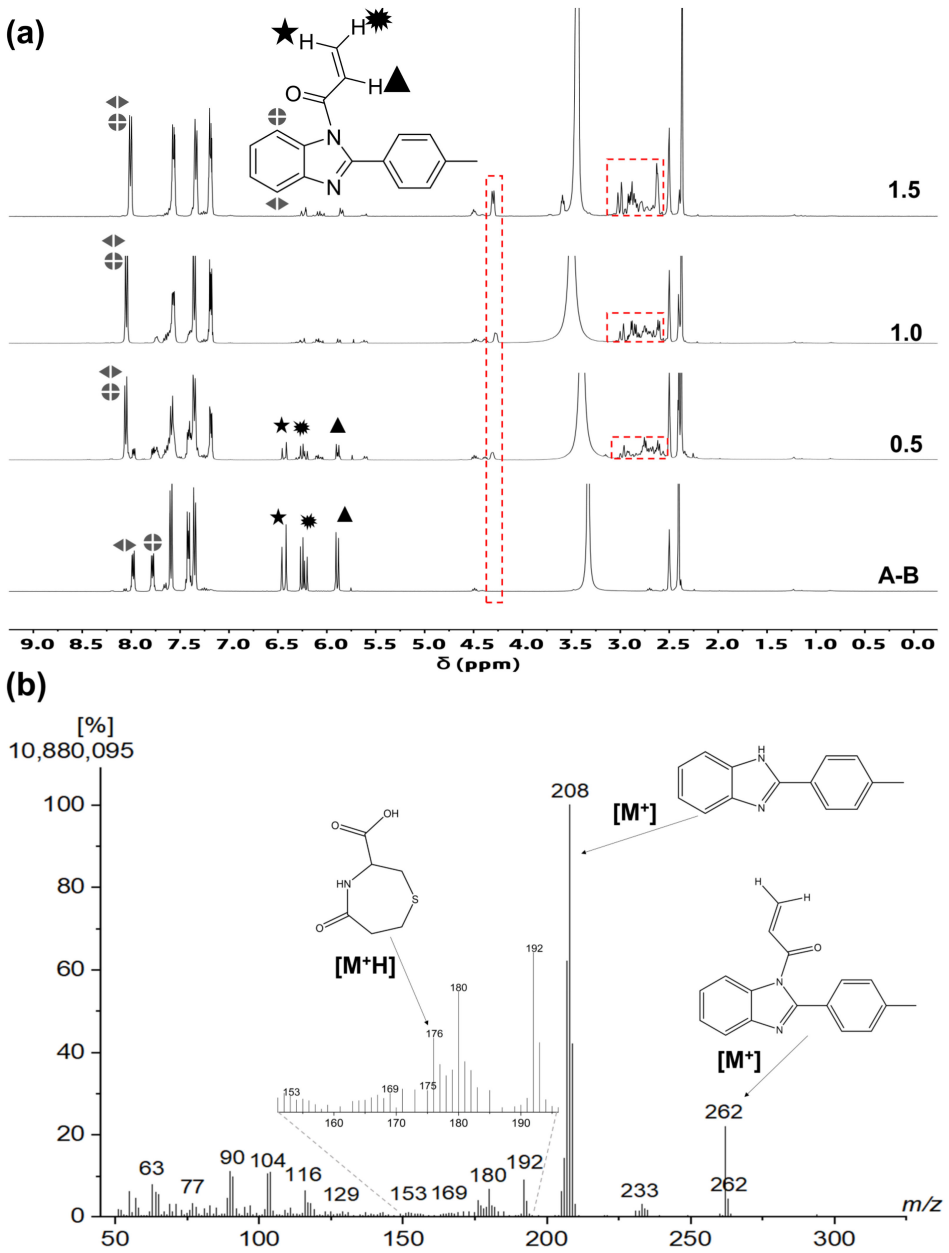
(**a**) ^1^H NMR spectra of **A-B** in DMSO-d6 after the addition of 0.5, 1, and 1.5 equivalents of Cys (red rectangles indicates protons for the seven-membered ring), (**b**) mass spectra of products obtained after equimolar reaction of **A-B** and Cys for 60 min at 25 °C.

The mechanism for detecting Cys by **A-B** was studied using ^1^H-NMR and mass spectroscopy. As shown in Figure 4, 0.5, 1.0, and 1.5 equivalents of Cys were serially added to the 90% DMSO:1.0 mM HEPES buffer solution (DMSO-d_6_ and D_2_O were used as solvent) containing **A-B**. Upon reaction of Cys with **A-B**, the peaks at 5.88, 6.20, and 6.42 ppm corresponding to the acrylate group completely disappeared upon the addition of 1.0 equivalents of Cys. At the same time, the peaks corresponding to the seven-membered ring product appeared at 2.60, 2.96, and 4.30 ppm. These results indicate that the mercapto group in Cys undergoes Michael addition on the acrylate group of **A-B**. Then, the intramolecular attack of the amine group of Cys on the carbonyl of **A-B** results in the removal of a cyclized product along with the formation of compound **3** (Scheme 2). The appearance of compound **3** after the reaction of **A-B** with Cys is also evidenced by the resultant fluorescence “turn-on” effect shown in Figure 3b.

Another piece of evidence for the Cys detection mechanism was deduced from the mass spectra of a reaction product obtained by reacting (60 min at 25 °C) equimolar **A-B** and Cys in a 90% DMSO:1.0 mM HEPES buffer solution. The solvent was removed at 40 °C under a high vacuum after the 60 min incubation. Then, the resultant residue was dissolved in methanol to prepare a sample for acquiring mass spectra. The obtained mass spectrum is presented in Figure 4b. The cyclization process of Cys after reaction with **A-B** generated a seven-membered ring (calcd. [H^+^]: 176.0376; found m/z: 176.0) and compound **3** (calcd. [H^+^]: 208.1000; found m/z: 208.10) upon deacrylation of **A-B**. Therefore, these results indicate the high applicability of **A-B** as a fluorescence turn-on sensor for detecting Cys in serum samples.

**Figure 5:**
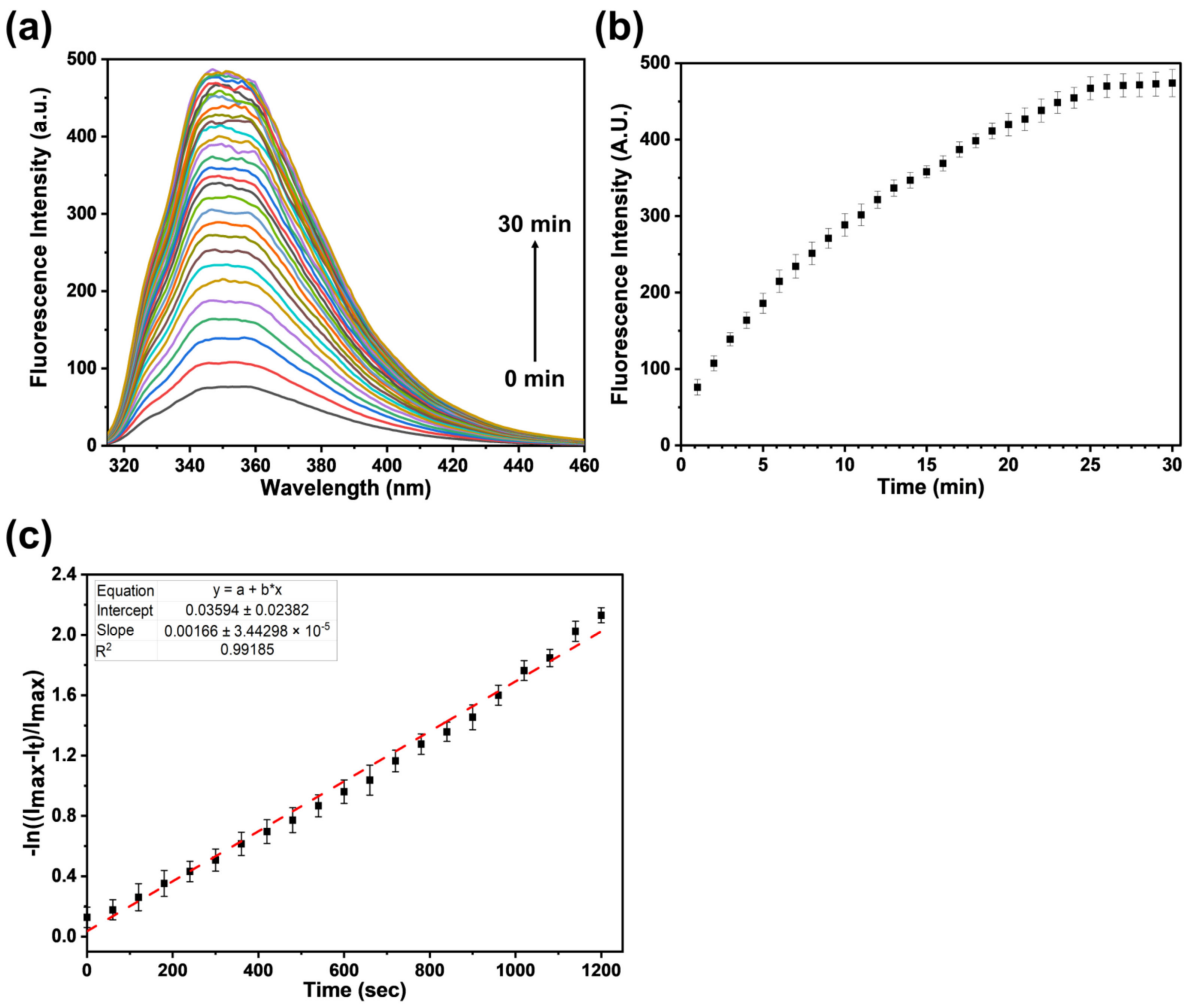
(**a**,**b**) Time-dependent fluorescence response and (**c**) pseudo-first-order kinetics plot for reaction of **A-B** (10 µM) with Cys (50 µM) in 0.01 M HEPES buffer solution (>0.1% DMSO) (λ_ex_ = 303 nm, λ_em_ = 350 nm) at room temperature.

As shown in Figure 5, we traced the time-dependent fluorescence intensity of **A-B** (10 µM) for 30 min upon adding two equivalents of Cys to estimate the minimum response time. The calculated pseudo-first-order rate constant for Cys was 1.66 × 10^−3^ s^−1^. These results indicate that probe **A-B** can detect Cys within 10 min.

### 3.3. Interference Study for the Detection of Cys by ***A-B***

Various amino acids, cations, and anions in the serum can interfere with the detection of Cys. One significant criterion for a highly functional probe is the ability to selectively detect the target analytes (i.e., Cys) with a specific response in the presence of a wide range of potentially competing analytes, including amino acids, and avoid cross-sensitivity. Therefore, for the clinical applicability of a fluorescent probe to detect Cys in serum samples, it is crucial to identify possible interfering agents. Hence, we studied the possible interference of various analytes on Cys detection using **A-B**. In competition experiments, the fluorescence spectra of **A-B** (10 µM) were recorded in the presence of 5 equivalents of Cys (50 µM) mixed with 5 equivalents of other analytes (50 µM).

**Figure 6:**
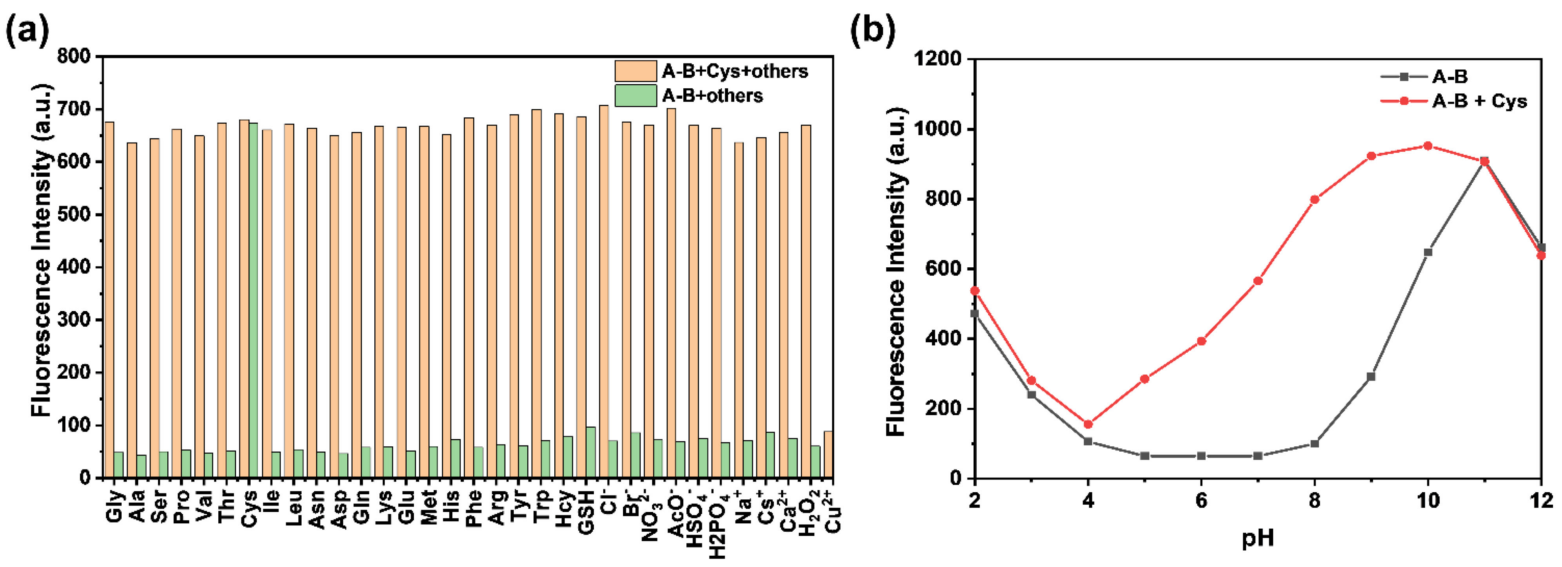
(**a**) Competitive study of **A-B** (10 µM) with and without the addition of five equivalents of various analytes (50 µM) in 1 mM HEPES buffer at room temperature, (**b**) effect of pH on the Cys detection (0, 50 µM) by **A-B** (10 µM) at various pH (λ_ex_ = 303 nm, λ_em_ = 350 nm).

As shown in Figure 6a, most of the tested analytes did not bring about the fluorescent variations in **A-B** resulting from the addition of Cys. Interestingly, the presence of an equimolar concentration of Cu^2+^ affects the detection of Cys among the tested biologically relevant analytes. It is well-known that the Cu^2+^ ions and Cys form a coordination complex through –SH and –COOH functional groups [48]. The complexation of Cys with Cu^2+^ inhibits the nucleophilic addition of –SH to the acrylate group. Even though the equimolar Cu^2+^ (50 µM) interfered in the Cys (50 µM) detection by **A-B** (10 µM) at studied conditions, it would not hinder detection in actual serum samples, as the level of Cu^2+^ (20 µM) [49] is negligible compared to Cys in healthy human serum (240–360 µM) [50,51,52]. Therefore, Cys detection by **A-B** in the actual serum samples would not be interfered with by Cu^2+^. These results expressly demonstrate that **A-B** could selectively detect Cys over other relevant species in the biological system.

### 3.4. Effect of pH on Cys Detection

The effect of pH (pH = 2–12) on Cys detection using **A-B** was examined with and without Cys (50 µM), as shown in Figure 6b. The fluorescence intensity of **A-B** without Cys did not show a significant change in the 4–8 pH range. This indicates that **A-B** has remarkable stability in this pH range. The fluorescence intensity increased in the presence of Cys from pH 4.0–12.0. It is important to note that below pH 8, –SH (*pKa* = 8.0) is the only nucleophile in Cys, as the *pKa* of α–NH_3_^+^ is 10.25. However, over pH 8, the α–NH_2_ acts as a nucleophile. Hence, both –SH and –NH_2_ groups in Cys react with **A-B** and enhance the deacrylation process with the increasing pH, resulting in higher fluorescence intensity. However, at pH 7, **A-B** demonstrated stabilized fluorescence signals in the presence and absence of Cys. Therefore, these results indicate that **A-B** is an excellent candidate for Cys detection in serum (pH = 7.4) and can be practically employed under physiological conditions.

### 3.5. Quantum Yield Measurement of Compound ***3***

The quantum yield of any fluorescence sensor is of paramount importance for its clinical application in detecting the analyte of interest. Here, though **A-B** is used to detect Cys, the resulting compound **3** after the reaction of **A-B** and Cys shows fluorescence. Hence, the quantum yield of compound **3** was determined using 2-aminopyridine (Φ = 0.60) as the standard [53]. We found that compound **3** demonstrates a quantum yield of 0.69. Therefore, we believe that **A-B** is an excellent fluorescence turn-on probe for detecting Cys in biological samples. Hence, we studied the applicability of **A-B** for Cys detection in the serum sample.

### 3.6. Calibration Curve and Determination of LOD

**Figure 7:**
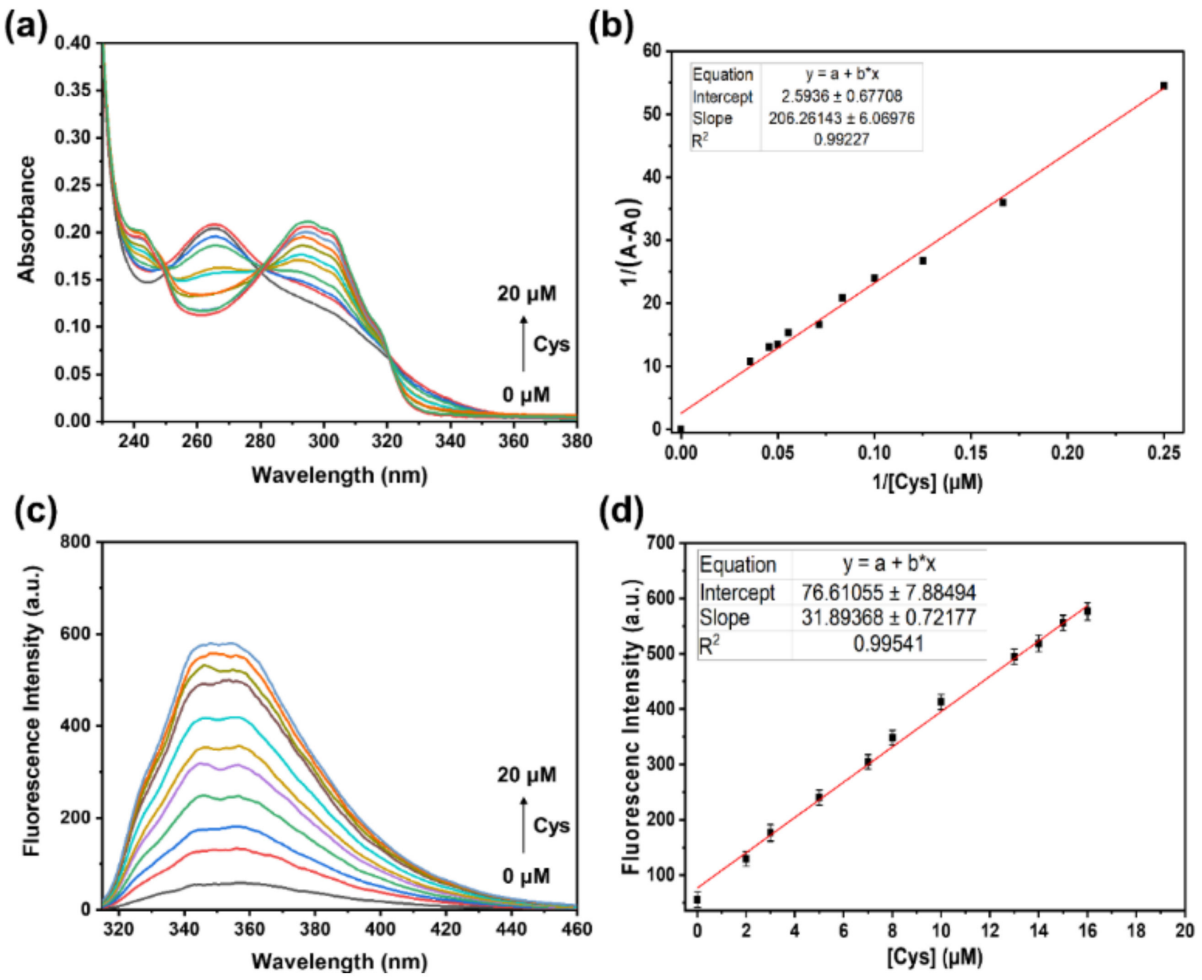
Changes in (**a**) UV–vis absorption spectra and (**c**) fluorescence spectra (λ_ex_ = 303 nm, λ_em_ = 350 nm) and respective calibration curves (**b**) and (**d**) for titration of **A-B** (10 µM) with Cys (0–20 µM) in 1 mM HEPES buffer at room temperature.

**A-B** (10 µM) was titrated with successive Cys (1 mM) additions in the final concentration range of 0–20 µM. The recorded UV−vis and fluorescence spectra are presented in Figure 7. The redshift in the UV-Vis absorption spectra of **A-B** was observed, as depicted in Figure 7a. The increase in absorbance as a function of increasing Cys concentration was plotted to obtain the standard curve presented in Figure 7b (λ_max_ = 303 nm). The LOD for detecting Cys by **A-B** using UV−vis spectroscopy was 86 nM. Similarly, changes in the fluorescence intensity with respect to added Cys were also recorded, as depicted in Figure 6b.

The fluorescence intensity of **A-B** at 350 nm was enhanced steadily with the incremental addition of Cys. The fluorescence intensity at 350 nm was plotted as a function of the Cys concentration to obtain a calibration curve, as shown in Figure 7d. The fluorescence titration data of **A-B** were used to calculate the LOD. The LOD was 43 nM and the linear detection range was 142 nM to 20 μM (limit of quantitation = 142 nM).

### 3.7. Detection of Cys by ***A-B*** in Serum Sample

The standard addition method for quantifying an analyte in serum samples allows investigating possible interference from serum matrix components. Therefore, we spiked the serum samples with Cys at various concentrations to validate the presented method. About 2 µL of these serum samples containing Cys was added to the solution containing **A-B** (10 µM) to obtain final concentrations of 2, 4, and 6 µM. The UV−vis and fluorescence spectroscopy measurements were performed to determine Cys levels using the respective standard curves (Figure 7b,d). The percent recovery and relative standard deviations were calculated as presented in Table 1. The linear regression coefficients of 0.99 with the recoveries from 94.5 to 110% for Cys measurement via the standard addition method confirm the possible clinical applications of **A-B**.

As presented in Table 2, there has been tremendous research on developing chemosensors for detecting Cys in various biological fluids, including serum. Several ring systems decorated with the reactive functional groups have been explored to generate a fluorescence turn-on probe selective to Cys. The most prevalent strategy among these methods is using the acrylic ester, established by Strongin’s group, to detect Cys over Hcy [40]. However, the choice of fluorophore is crucial considering the requisite application of the designed probe. Here, **A-B** was designed solely for detecting Cys in serum samples. Hence the simplest possible fluorophore, a benzimidazole scaffold, was used in this study. Unlike the reported probes presented in Table 2, synthesis of benzimidazole scaffold and subsequent modification with the acrylic ester to generate **A-B** are relatively easy as milder reaction conditions are required.

Further, the aqueous solubility of the probe is crucial for its applicability in detecting the Cys in serum samples. Most of the reported probes in Table 2 require an organic component in an aqueous solution to detect the Cys in biological fluids, limiting their clinical applicability. **A-B** successfully detects Cys in serum samples with comparable to significantly higher (2.3–5.4 fold) LOD than reported methods.

## 4. Conclusions

In conclusion, a fluorescence turn-on probe **A-B** was designed and synthesized using the benzimidazole scaffold. **A-B** was investigated for its ability to recognize Cys in the presence of various analytes, including other amino acids, anions, and cations. Endorsing the addition followed by elimination through cyclization of Cys in a stoichiometric ratio of 1:1 with **A-B** displayed high sensitivity and selectivity for Cys. The mechanism of Cys detection by **A-B** was fully validated using ^1^H NMR and mass spectroscopy. **A-B** with a quantum yield of 0.69 and almost no interference from the components in the serum matrix demonstrated high applicability for Cys detection in serum samples. **A-B** showed high selectivity and high sensitivity for Cys. **A-B** demonstrated a detection limit of 86 nM and 43 nM in HEPES buffer using UV−vis and fluorescence spectroscopy, respectively. The results presented here suggest high applicability of **A-B** in clinical settings for detecting and measuring Cys in serum samples.

## Figures and Tables

**Table 1:** Results of spiking test for Cys detection in human serum using **A-B**.

Analysis Method	[Cys] (μM)			
	Spiked	Found	Recovery (%)	RSD (%) (n = 3)
Fluorescence spectroscopy	2	2.20	110.1	0.10
	4	3.80	94.5	0.19
	6	5.96	99.3	0.11
UV−vis spectroscopy	2	2.30	114.4	0.06
	4	4.24	106.1	0.10
	6	6.31	105.2	0.21

RSD, relative standard deviation.

**Table 2:** Comparison of sensing behavior of present work with the reports in the literature.

Probe	LOD (nM)	Solvent System	Method	Ref.
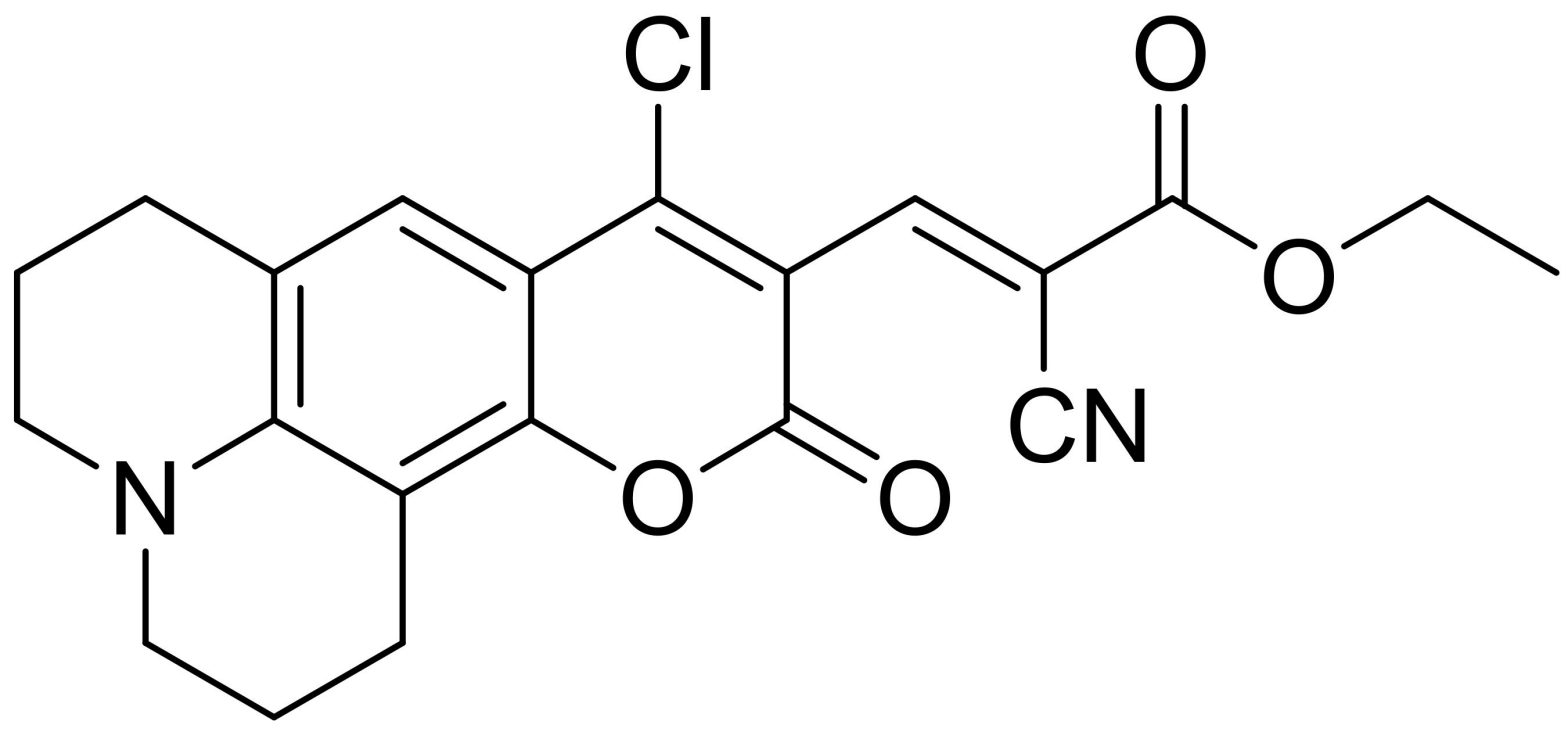	0.2	DMSO: PBS buffer (40:60)	Fluorescence	[54]
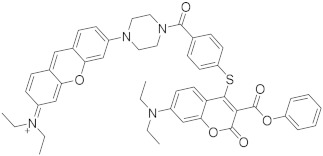	12	DMF: PBS buffer (30:70)	Fluorescence	[55]
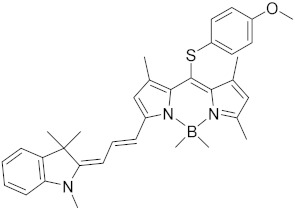	118	CH_3_CN	Fluorescence	[56]
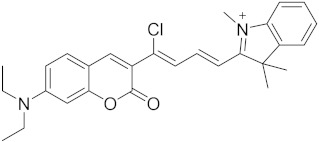	228	PBS	Fluorescence	[57]
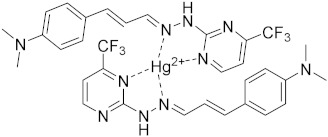	100	Bis-tris buffer	UV−visible	[58]
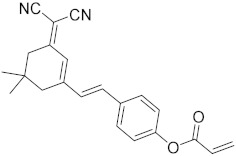	170	PBS buffer:CH_3_CN (80:20)	Fluorescence	[59]
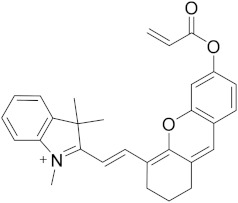	160	HEPES buffer	Fluorescence	[60]
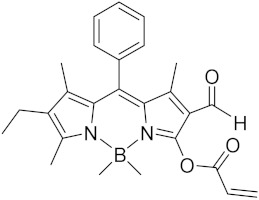	233	PBS buffer:CH_3_CN (50:50)	Fluorescence	[61]
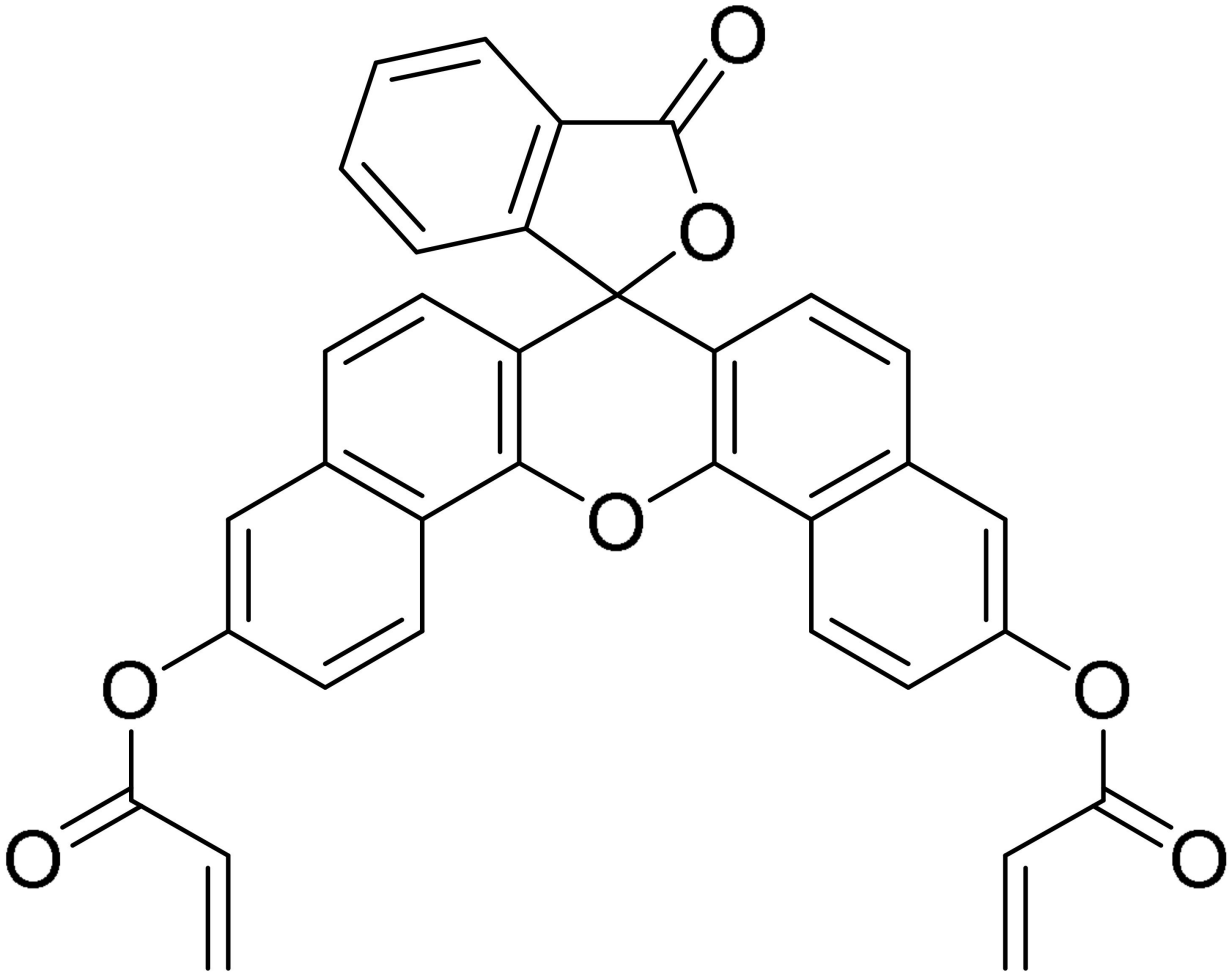	180	PBS buffer: DMSO (60:40)	Fluorescence	[62]
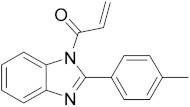	43	HEPES buffer	Fluorescence	-

## Data Availability

The data presented in this study are available upon request from the corresponding author.

## Supplementary Materials

The following supporting information can be downloaded at: https://www.mdpi.com/article/10.3390/bios12040224/s1. Figure S1: ^1^H-NMR spectrum of compound **3**, Figure S2: ^13^C NMR spectrum of compound **3**, Figure S3: Mass spectrum of compound **3**, Figure S4: ^1^H-NMR spectrum of **A-B**, Figure S5: ^13^C NMR spectrum of **A-B**, Figure S6: Mass spectrum of **A-B**, Figure S7: Image taken under UV lamp depicting the selectivity of **A-B** for Cys among other amino acids. The addition of Cys (50 µM) to the solution of **A-B** (10 µM) results in fluorescence turn-on effect.
