# Supplementary material for: Elimination Reaction-Based Benzimidazole Probe for Cysteine Detection and Its Application in Serum Sample Analysis
Source: Biosensors (Basel). 2022 Apr 8;12(4):224. doi: 10.3390/bios12040224 (PMC9031725; doi:10.3390/bios12040224)
Supplement: Supplementary file 1 [file biosensors-12-00224-s001.zip › biosensors-1634298-supplementary.pdf]

Elimination Reaction-Based Benzimidazole Probe for Cysteine Detection and Its Application in Serum Sample Analysis

In-ho Song [1,†], Gyu Seong Yeom [1,†], Anil Kuwar [2] and Satish Balasaheb Nimse [1,*]

[1] Institute of Applied Chemistry and Department of Chemistry, Hallym University, Chuncheon 24252, Korea; songin-ho@hallym.ac.kr (I.-h.S.); m21019@hallym.ac.kr (G.S.Y.)
[2] School of Chemical Sciences, KBC-North Maharashtra University, Jalgaon 425001, India; kuwaras@gmail.com
* Correspondence: authors: satish_nimse@hallym.ac.kr
† These authors contributed equally to this work.

Supplementary Information

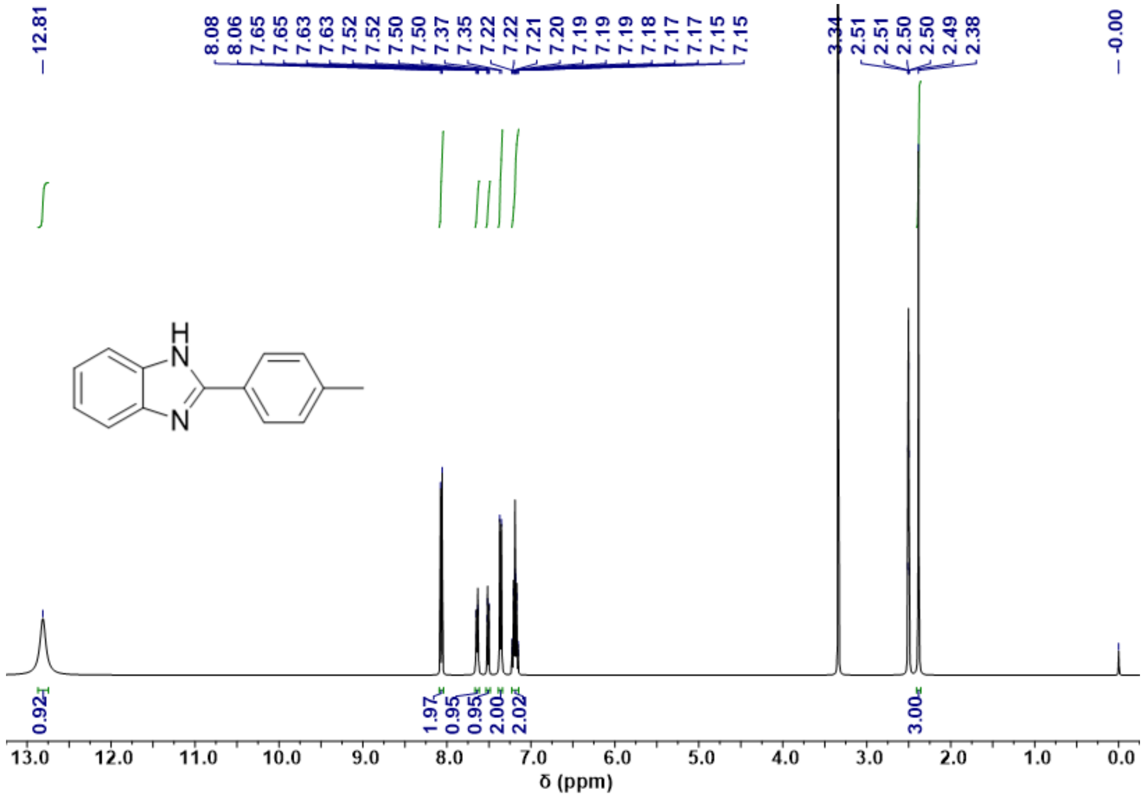

Figure S1.^{1}H-NMR spectrum of compound **3**.

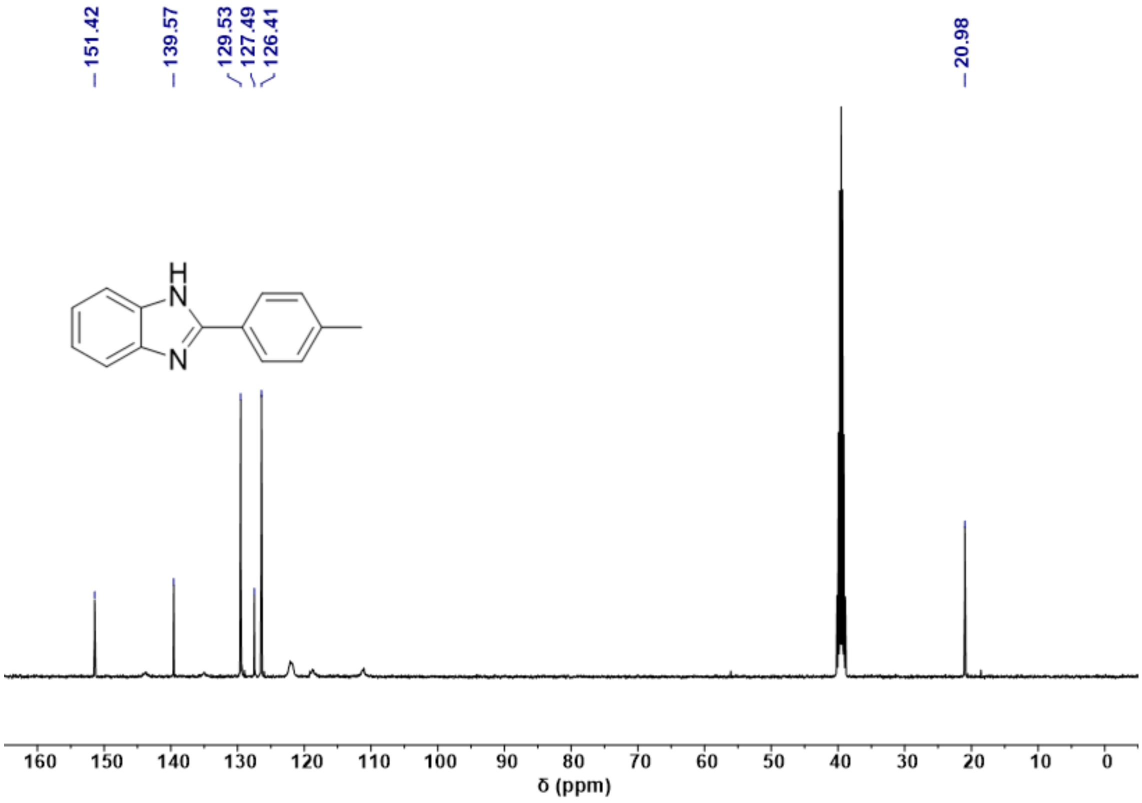

Figure S2. ^{13}C NMR spectrum of compound **3**.

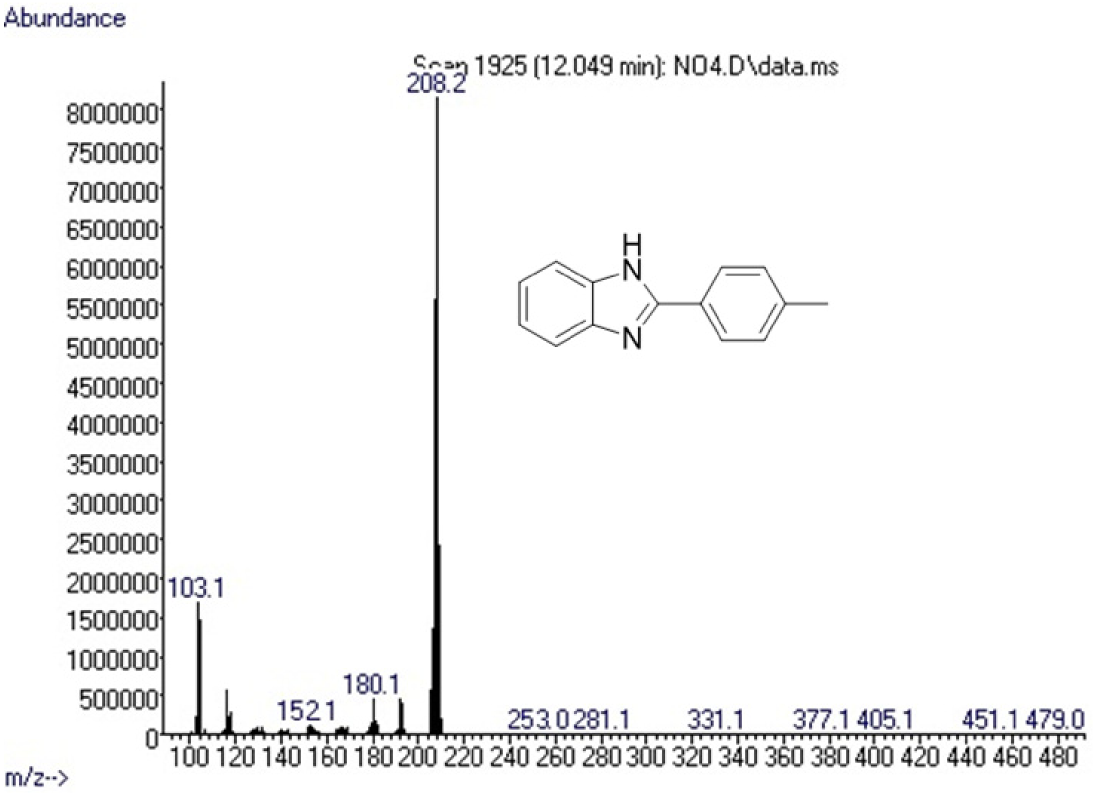

Figure S3. Mass spectrum of compound **3**.

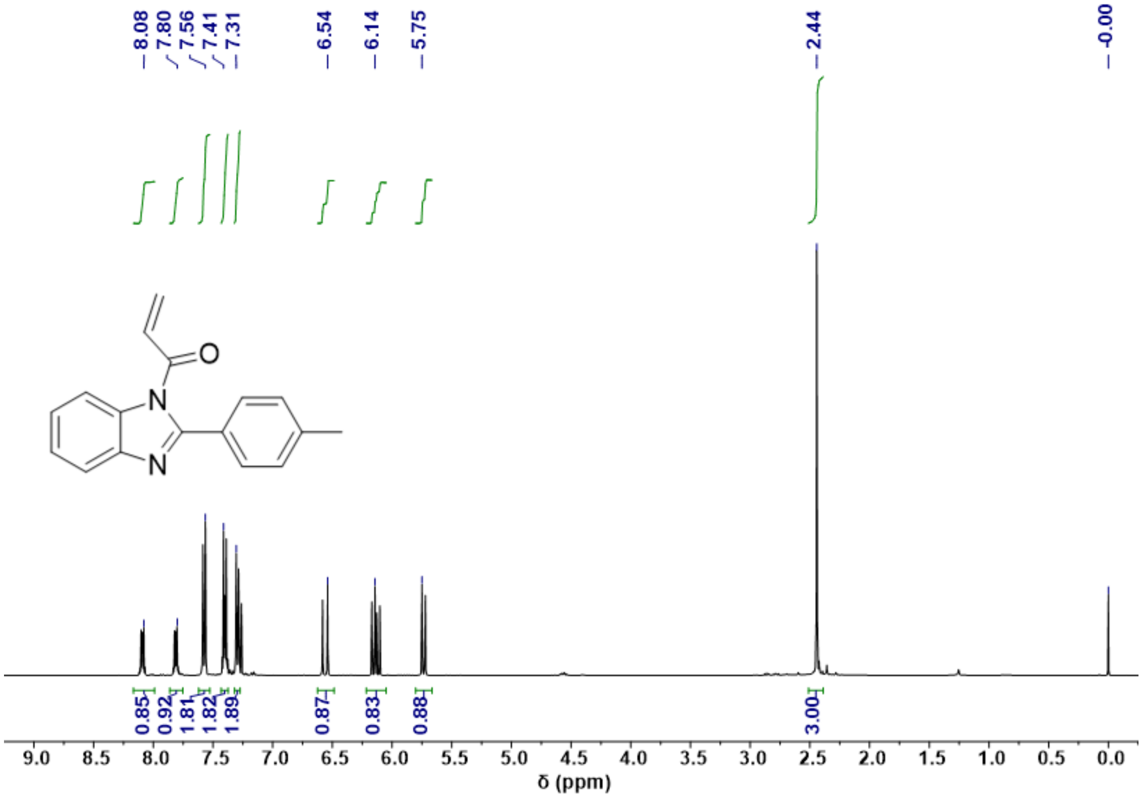

Figure S4.^{1}H-NMR spectrum of **A-B**.

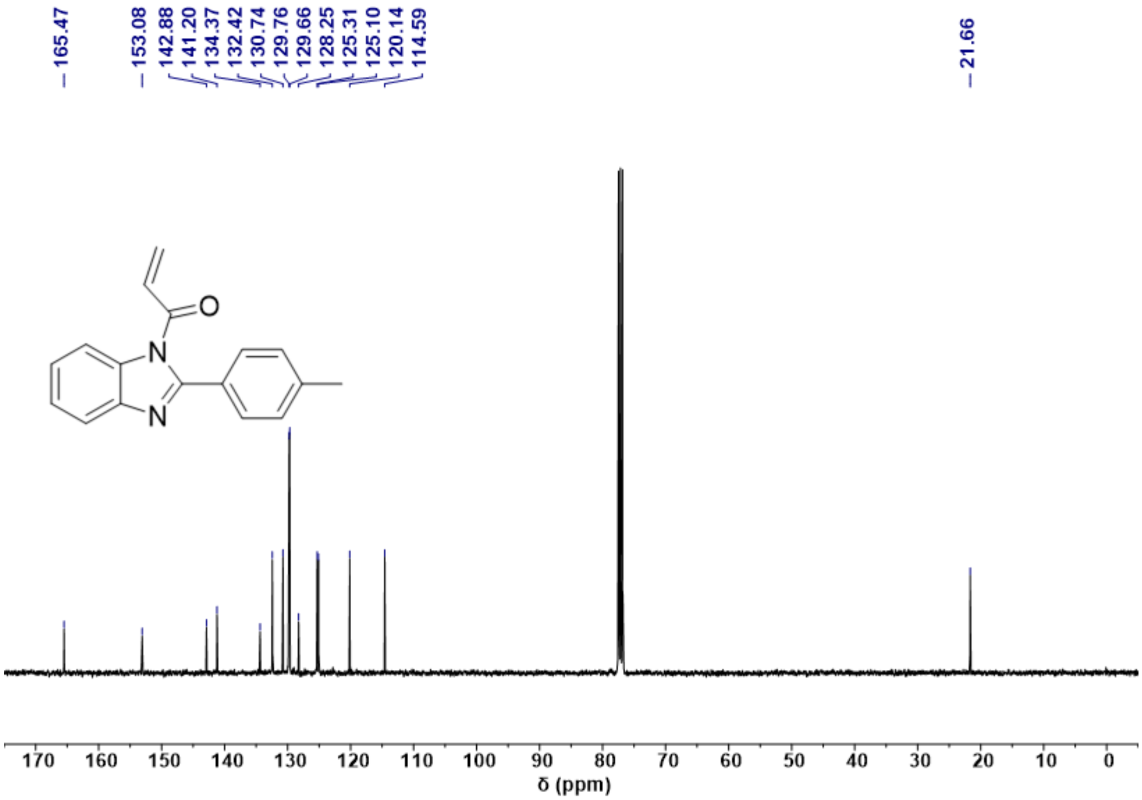

Figure S5. ^{13}C NMR spectrum of **A-B**.

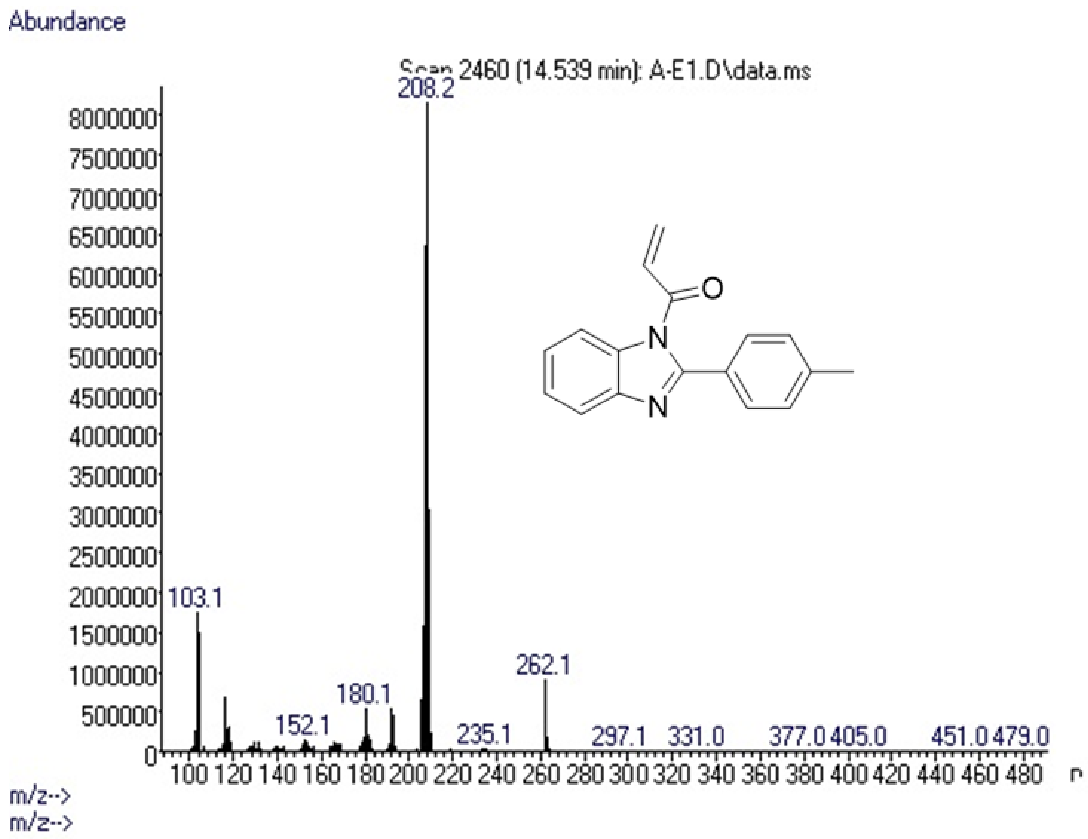

Figure S6. Mass spectrum of **A-B**.

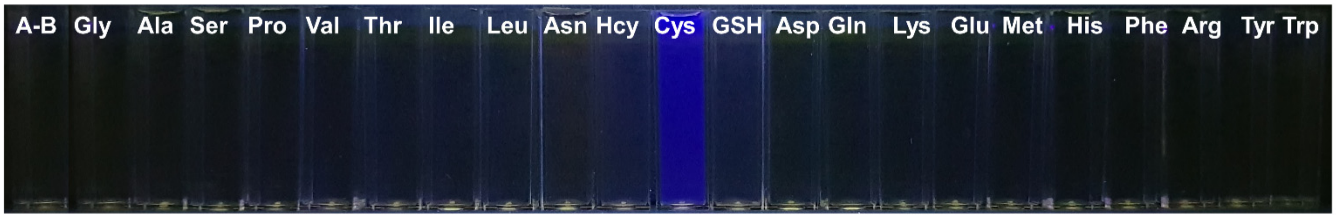

Figure S7. Image taken under UV lamp depicting the selectivity of **A-B** for Cys among other amino acids. The addition of Cys (50 μM) to the solution of **A-B** (10 μM) results in fluorescence turn-on effect.
